# Home food preparation practices, experiences and perceptions: A qualitative interview study with photo-elicitation

**DOI:** 10.1371/journal.pone.0182842

**Published:** 2017-08-30

**Authors:** Susanna Mills, Martin White, Wendy Wrieden, Heather Brown, Martine Stead, Jean Adams

**Affiliations:** 1 Institute of Health & Society, Newcastle University, Newcastle upon Tyne, United Kingdom; 2 Centre for Diet and Activity Research (CEDAR), MRC Epidemiology Unit, University of Cambridge School of Clinical Medicine, Cambridge, United Kingdom; 3 Human Nutrition Research Centre, Institute of Health & Society, The Medical School, Newcastle University, Newcastle upon Tyne, United Kingdom; 4 Institute for Social Marketing, University of Stirling, Stirling, United Kingdom; Universidade Federal de Minas Gerais, BRAZIL

## Abstract

Food-related choices have an important impact on health. Food preparation methods may be linked to diet and health benefits. However, the factors influencing people’s food choices, and how they are shaped by food preparation experiences, are still not fully understood. We aimed to study home food preparation practices, experiences and perceptions amongst adults in North East England. A matrix was used to purposively sample participants with diverse socio-demographic characteristics. Participants developed photographic food diaries that were used as prompts during semi-structured interviews. Data were analysed using the Framework Method. Interviews were conducted with 18 adults (five men and 13 women), aged approximately 20 to 80 years, to reach data saturation. Participants’ practices varied widely, from reliance on pre-prepared foods, to preparing complex meals entirely from basic ingredients. Key themes emerged regarding the cook (identity), the task (process of cooking), and the context (situational drivers). Resources, in terms of time, money and facilities, were also underpinning influences on food preparation. Participants’ practices were determined by both personal motivations to cook, and the influence of others, and generally reflected compromises between varied competing demands and challenges in life. Most people appeared to be overall content with their food preparation behaviour, though ideally aspired to cook more frequently, using basic ingredients. This often seemed to be driven by social desirability. Home food preparation is complex, with heterogeneous practices, experiences and perceptions both between individuals and within the same individual over time, according to shifting priorities and circumstances. Generalisability of these findings may be limited by the regional participant sample; however the results support and build upon previous research. Focussing interventions on life transition points at which priorities and circumstances change, with careful targeting to stimulate personal motivation and social norms, may prove effective in encouraging home food preparation.

## Introduction

Food choices, including meal source and preparation method, have an important impact on dietary intake, and hence health. Preparing food at home has been associated with a range of potential benefits, such as consuming fewer calories and smaller portions, and eating less fat, salt and sugar.[1, 2] Home food preparation is also positively correlated with greater intake of fruits and vegetables[3] and a healthful dietary pattern.[4] Recent systematic reviews have identified potential advantages of home cooking interventions, in terms of diet, health, and cooking knowledge/skills, confidence and attitudes.[5, 6] However, they also found the evidence base was overall inconclusive, due to the predominance of poor quality studies.[5, 6] Cooking classes for children, parents and carers have been recommended as part of wider strategy to reduce childhood obesity[7].

It is estimated that by 2020, non-communicable diseases (NCDs) will account for 60% of all disability adjusted life years and nearly 75% of all deaths worldwide,[8, 9] with the majority of NCDs related to diet.[8] In almost every part of the world, health problems attributable to NCDs associated with dietary intake now outweigh the burden due to undernutrition[8, 10–12].

There are no standardised, widely accepted definitions for home cooking and food preparation.[13] The terms are used here interchangeably, to refer to making food ready to eat. However, cooking is generally used here in the context of meals, whereas food preparation includes less structured eating occasions such as snacks.

Internationally, a perceived decline in cooking skills has been reported by food and nutrition practitioners, policy makers and scientists,[13–15] although some evidence suggests that skill deficits may be restricted to particular population subgroups.[16] The frequency and amounts of time spent on home food preparation using basic and raw ingredients in the United Kingdom have also been declining, in comparison with other countries such as France[17].

Qualitative research into home food preparation is likely to be particularly insightful for exploring the nuances of this contextualised and highly individual behaviour. A recent systematic review identified only 11 qualitative studies with a main focus on the determinants and/or outcomes of home cooking.[18] In general, studies sought information solely through traditional interview or focus group methods, which can have limited capacity to generate rich, insightful data regarding everyday practices that are often undertaken with minimal reflection.[19] The studies also usually considered only one aspect of cooking behaviour and did not describe in detail the rationale for and experiences of decisions relating to different approaches to cooking. Most studies focussed on a specific demographic group, such as the experiences of working mothers,[20] or a particular social context, for example acculturation following immigration[21].

Similar data from participants with wide-ranging socio-demographic characteristics would help inform development of public health interventions to encourage home cooking, and enhance understanding of the broad range of factors influencing behaviour. Further research to explore the nature and perceptions of home cooking practices has been advocated.[22] Contemporary studies are particularly important in view of the rapid evolution of influential social and economic determinants. These include increasing female participation in the workforce,[23] growing domination of large supermarkets in the grocery market,[24] and increasing availability of pre-prepared meal options.[25]The aim of this study was to explore the practices, experiences and perceptions of home food preparation amongst adults in North East England, in order to identify the key themes of public health importance, traversing diverse socio-demographic characteristics and social circumstances. This aim was successfully achieved through qualitative interviews with photo-elicitation.

## Materials and methods

### Participants and recruitment

This study adhered to the COREQ consolidated criteria for reporting qualitative research.[26] We undertook semi-structured interviews with photo-elicitation to explore home food preparation behaviour. The majority of interviews were one-to-one; however for three interviews, two of the other research participants were also present, in accordance with the participants’ requests. These participants were all known to each other, had consented to take part in the research, and contributed to the interview dialogue.

We purposively recruited adult participants from the North East of England between June and October 2015, through social media advertisements, voluntary organisations, academic recruitment networks, and health, employment and community groups. We used a sampling matrix to ensure diverse participant representation according to gender, age, ethnicity, marital status, household composition, deprivation, self-reported weight status, and self-reported interest and skills in cooking. Area based deprivation was measured using the 2015 index of multiple deprivation (IMD), assigned to unit postcodes and allocated to fifths of the distribution.[27] The aim was not to recruit a sample that met all possible combinations in the matrix, but rather to interview participants with diverse characteristics, in order to identify key issues of public health importance. Individuals aged less than 16 years, and those who were not the main or shared main household food provider as defined previously[28] were excluded, since they were anticipated to have fewer insights to contribute towards the research questions.

Depending on the recruitment method, either the potential participant saw advertising material and contacted the researcher to express their interest, or the participant responded to the researcher directly, following an in-person promotional presentation to a group. Participants were met on two occasions by SM, a female doctoral researcher who is qualified as a medical doctor and has a background in public health. SM received prior in-depth training in qualitative research methods and analysis.

At the first meeting, the participant information sheet was reviewed, and the participant was provided with the opportunity to ask any outstanding questions, before completing the written consent form. Participants were asked to take photographs, which they would then present and discuss at interview.[29, 30] The researcher explained this process, and asked the participant to submit at least one digital photograph via email each day, over the period of one week. Participants were encouraged to photograph all aspects of food and eating at home, such as food shopping, cooking and eating facilities, and mealtimes. For participants who did not own a smartphone with capacity to take and send photographs, a digital camera was provided, and photographs were uploaded and sent by computer. In order to maintain anonymity, participants were advised to avoid taking identifiable images of people. A daily text message reminder service was offered.

### Data collection

Interviews were conducted one week after the initial meeting, at the participant’s home; Newcastle University; or a public venue such as a local community centre. There was no relationship between the participants and the researcher before the study started. Research participants were aware that the interviewer was a medical doctor, but that the focus of the study was not to provide a critique of their diet, nor to offer medical advice.

Interviews followed a semi-structured interview topic guide with largely open-ended questions (see version 1 topic guide in S1 Appendix). This was informed by a recent extensive systematic review of the barriers and facilitators of home cooking,[18] and piloted. In the main interviews, some questions were expanded and iteratively developed as the study progressed, according to previous participants’ responses, as previously[31].

We used the process of photo-elicitation to generate additional participant data and provide a form of visual diary to prompt in-depth interview responses. Interviews commenced by asking each participant to present and discuss their photographs of food and eating. Two participants did not take any photographs and therefore this stage was omitted. Participants were encouraged to ‘tell their story’ of home food preparation, and questions from the topic guide were used to probe emerging themes and concepts further (see S1 Appendix). Interviewing and concurrent data analysis continued to data saturation, whereby existing themes were consistently repeated, and no new themes emerged from the data.[32] All interviews were audio-recorded, transcribed verbatim, and anonymised; basic field notes were made at the time of the interview. Transcripts were not returned to participants for comment; however all participants were invited to receive a copy of the research findings at the end of the study if they so wished.

### Analysis

We analysed interviews using Framework Analysis,[33] focussing particularly on emergent key public health issues. Framework Analysis provides the benefit of a systematic approach to comparing inter- and intra- participant viewpoints, and entails coding data according to the salience of emerging themes and concepts, rather than their frequency of occurrence[34].

NVivo 10 software was used to manage the data, using Framework Analysis in a stepped process.[33] In step 1, we reviewed initial transcripts, and recorded key ideas and recurrent themes regarding home food preparation. In step 2, a provisional thematic framework was constructed, incorporating themes highlighted from previous research[18, 35] and key themes from step 1, and directed by the research aims. Subsequently, in step 3 we applied the thematic framework to successive interviews, thereby facilitating simultaneous data collection and analysis. The framework was modified and iteratively expanded to incorporate new emerging themes and ideas, including participants’ approaches to photo-elicitation. In step 4, we charted data according to themes using Microsoft Excel, to enable comparisons within and between participants. Finally, step 5 involved exploring further relationships, patterns and associations within the data, including emerging overarching concepts and principles.

The whole research team (SM, JA, MW, WW, HB and MS) were involved in the development and review of data analysis. MS has extensive experience in conducting and analysing qualitative research. The lead researcher (SM) coded the dataset independently and iteratively to develop a set of key themes. A subset of transcripts (n = 3) were discussed in a data clinic with other members of the research team early in the analysis phase, to review the interpretation of emergent perspectives and themes. A further subset (one transcript each, for three members of the research team) was coded independently using the final coding frame, to check the reliability of the coding process. SM attended a departmental qualitative data sharing group to improve understanding and experience of interpreting themes, at which she presented and received feedback on her interpretation of the interview data.

### Ethics

This research was approved by the Newcastle University Faculty of Medical Sciences Research Ethics Committee, application number 008585 2015. All participants submitted informed, written consent prior to taking part in the study. After the interviews were completed, the interviewer provided a debriefing sheet and reiterated data management, confidentiality, and use of data in research and publications. Participants were able to ask any outstanding questions and received a £20 shopping voucher as reimbursement for their time, as advertised.

## Results

We recruited a total of 19 adult participants to the study; one participant withdrew after the first meeting, leaving 18 participants’ data for analysis. Characteristics of those taking part are shown in Table 1.

**Table 1:** Characteristics of interview participants.

Participant	IMD fifth	Gender	Ethnicity	Age (years)	Marital status	Living with	Weight	Interest in cooking	Standard of cooking
1	3rd	Female	White British	≤30	Single, cohabiting	Partner	Overweight	High	High
2	3rd	Female	White British	31–45	Single, cohabiting	Partner	Overweight	High	Medium
3	1st	Male	White British	56–65	Married	Partner	Normal	Low	Low
4	5th	Male	White British	31–45	Single	5 unrelated people in shared house	Normal	Medium	Medium
5	2nd	Male	White British	≥66	Divorced	Alone	Normal	High	Medium
6	1st	Female	White British	31–45	Married	Partner and 2 children	Overweight	High	High
7	5th	Female	Pakistani	31–45	Married	Partner and 2 children	Normal	Medium-high	High
8	2nd	Male	White British	≥66	Widower	Alone	Overweight	Low	Low
9	3rd	Male	White British	≥66	Divorced	Part-time living-in partner	Normal	Low	Low
10	5th	Female	White British	31–45	Single, cohabiting	Partner and 2 children	Overweight	High	Medium-high
11	5th	Female	White British	31–45	Single, cohabiting	Partner and 2 children	Overweight	Medium	Medium
12	5th	Female	White British	31–45	Single	3 children	Normal	High	Medium
13	5th	Female	Black African	31–45	Single, cohabiting	Partner and 1 child	Overweight	High	High
14	5th	Female	White British	≤30	Single (engaged)	Mother (full time live-in carer)	Overweight	High	Medium
15	4th	Female	White British	31–45	Single	Alone (partner lives in flat upstairs)	Overweight	Low	Low
16	5th	Female	Bangladeshi	31–45	Married	Partner and 2 children	Normal	High	High
17	5th	Female	White British	31–45	Married	Partner and 2 children	Overweight	Low-medium	Medium
18	5th	Female	White British	≤30	Single	1 child	Normal	Low	Low
19 (withdrew)	3rd	Female	White British	≤30	Single, cohabiting	Partner and 1 child	Overweight	Medium	Low

Index of Multiple Deprivation (scale 1 to 5: 1 = least, 5 = most deprived fifth of distribution). Interviews 14, 15 and 16 were shared interviews. Weight: self-reported as underweight/normal/overweight; Interest in cooking and standard of cooking: self-reported as: low/medium/high

Interviews lasted between 36 minutes and 1 hour 18 minutes. We present key underpinning principles identified from the research, then describe the main emergent themes using supporting quotations, referring to illustrative participant photographs where applicable (photographs shown in S2 Appendix). The number of photographs participants submitted (range 1 to 97), and their choice of material, varied greatly. Some participants systematically photographed all meals and eating occasions daily, whereas others selected images to illustrate habits or deviation from usual practices. This variation complemented the range of perspectives and experiences of home food preparation that participants described during interviews. Longer interviews generally corresponded to larger collections of participant photographs submitted for discussion.

With regards to interview findings, overall participants viewed cooking as a balance between varied competing influences and demands in life. Most people appeared to have the essential resource requirements, such as time and money, necessary to reach a level of compromise in cooking with which they were generally content. Many participants described strategies they had adopted to juggle an aspiration to regularly cook healthy meals on the one hand, with the challenge of fitting food preparation conveniently into busy lives on the other. Often people seemed to conclude that under perfect conditions they would aim to cook more often, and use basic ingredients more extensively. But given other competing demands, they were comfortable to make compromises. For those participants who aspired to change, this was apparently often driven by social desirability to prepare more complex, healthy meals for themselves and others, and the fulfilment of an ideal or self-identity as a competent cook.

*I would like it to be different in the sense that I would like to feel that I could give myself the time to do it [cooking] and enjoy it*. *But that feeling isn’t strong enough to make it happen*, *because there is always something that I would rather be doing*. *PARTICIPANT 9* (see photograph 1)

**Fig 1:**
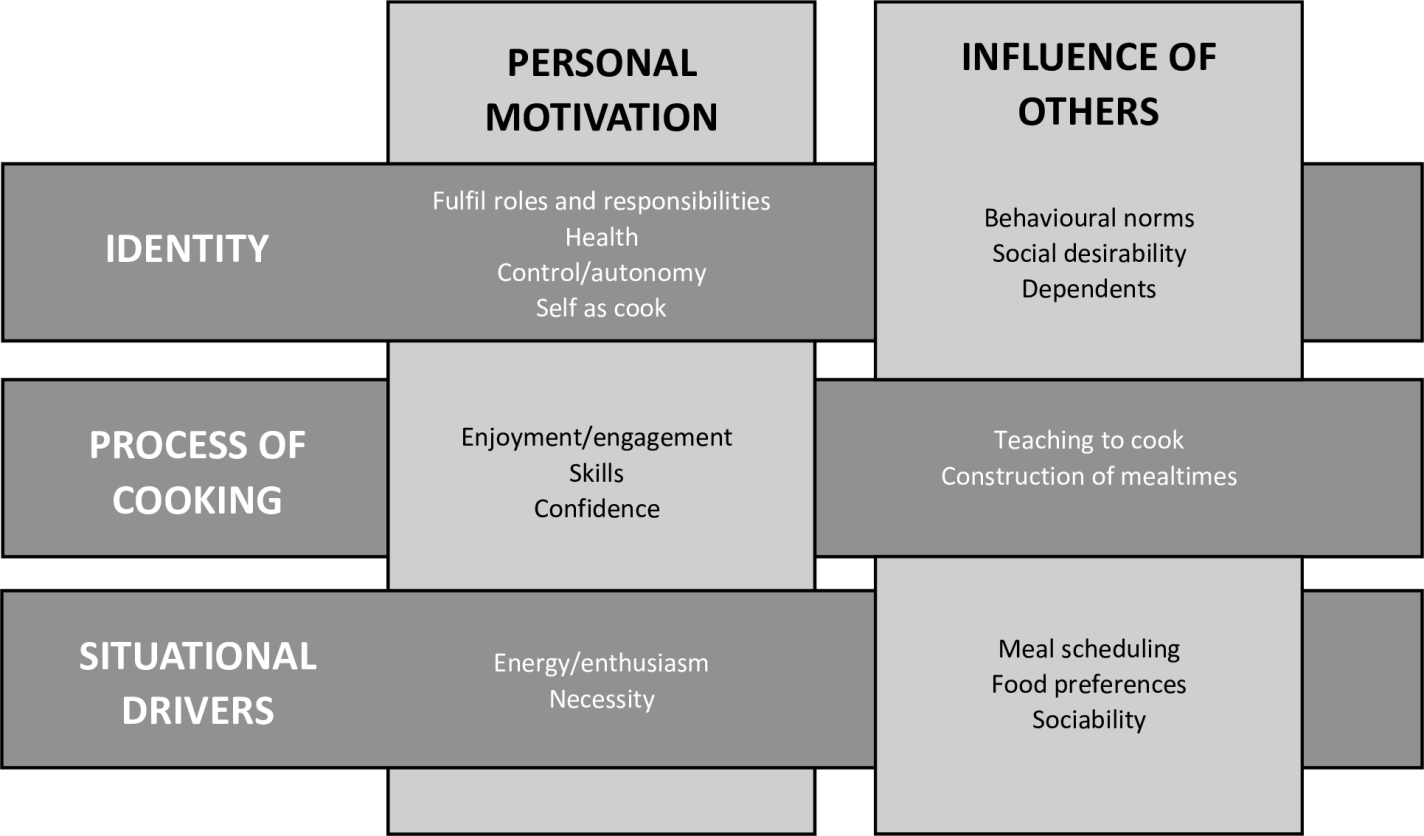
Matrix of main interview themes. Terms in smaller type indicate concepts that determined participants’ home food preparation behaviour, categorised by one of three themes, and one of two sources of motivation. For example ‘fulfil roles and responsibilities’ provided a personal motivation to cook, and was recognised as part of the participant’s identity. Underpinning all themes was a consideration for resources, namely time, money and facilities.

The main emergent interview themes are depicted in Fig 1. We identified three key themes regarding home cooking in terms of the cook (identity); task (process of cooking); and context (situational drivers). These were each shaped by both personal motivation, and the influence of others; these associations were fluid, with overlap and inter-relationships between categories. A fourth theme of resources, with consideration for time, money, and facilities, straddled these concepts. The relationships between these themes are explored further below.

### Identity

For many participants, the roles and responsibilities they had currently adopted in life provided a key personal motivation to cook. For example, several women perceived that part of their duty as a mother and homemaker was to provide meals, particularly those that were healthy and nutritious, for the household. Similarly, some participants described motivation to cook in their role as spouse or carer. This sense of responsibility was often persistent, shifting only at different life transition points as participants’ living context and roles changed with time, and could override more transient levels of energy and enthusiasm.

Well when my wife was at home, which she was for some time after she took ill, I did make an effort and cooked things which I thought she would eat, because she wasn’t eating very well… And there was the incentive to do it then because I was doing it for her. PARTICIPANT 8

Many participants also recognised that they were influenced by others, through behavioural norms and social desirability around providing home cooked meals for dependents. In particular, participants often seemed embarrassed about serving meals straight out of a packet, without any personal contribution.

*I am at home so for me this has been my setting*. *This is a role that I’ve taken on*, *so I’m the main homebody in this home*, *so for me cooking and having the cleaning and everything done*, *that in a sense is a mother’s role*, *but that’s how I feel it is*. *PARTICIPANT 7* (see photograph 2)

*I might buy something like these lamb kebabs which if you buy all the component ingredients that are pre-prepared it’s not like–I like to think it’s like the next step up from a ready-meal*, *if you like*… *So it’s not like a meal out of a pot ready*, *all-in-one*. *It’s a meal that you’ve put together but it’s really convenient… PARTICIPANT 2* (see photograph 3)

For many participants, their own health and that of dependents was influential. Most people were aware of healthier foods and cooking methods, and tried to choose these when possible, particularly in view of existing health conditions. Participants were generally in agreement that preparing food at home was a healthier choice than alternatives such as pre-prepared foods and take-aways.

Well they’re unhealthy [take-aways], and when I went to this seminar for my gastric band they showed you how, like they’d done a national survey and they showed you how many calories and stuff there was in them and I was, like, ‘and how much sugar’? There was ninety grams of sugar in a Korma and I was, like, ‘What?’ PARTICIPANT 14

Some participants described growing in confidence with cooking over the life course, which enabled them to develop their own self-identity as a cook, and exercise autonomy. One participant commented that whilst in the past she was always trying to imitate others, over time she established her own signature dishes.

*Because to me it’s part of being independent*, *you make your own food*, *you clear up after yourself*, *that kind of thing*. *PARTICIPANT 4* (see photograph 4)

### Process of cooking

Many participants recognised that they were inherently interested in food and cooking and enjoyed the activity, or to varying degrees were disinterested and disliked it. Frequently, personal interest in food and cooking appeared to last lifelong. However, some individuals reported changing levels of enthusiasm and engagement at different stages in life, as new roles or influences became important.

But the longer we were together the more interest I took in making meals. And when we had the children we couldn’t go out for ages because they were quite close together, and so I used to watch a lot of cookery programmes when I was off on maternity leave, and try things out. And like I say having family over you feel the need to make an effort. So I really came to love doing it, and liked to read recipes and, you know, it’s nice… So I have grown into it definitely. PARTICIPANT 6

*Well*, *I don’t like cooking*… *And*, *in fact*, *I don’t do it*… *So if I eat in*, *it is inevitably a frozen meal*… *Microwaves are very handy*… *And that’s it*. *PARTICIPANT 8* (see photograph 5)

A few participants reported drawing inspiration for their cooking from television programmes; however some also noted that in their household, those who prepared food the least frequently were also the most likely to enjoy watching cookery shows. Participants often described learning how to cook from other people, frequently relatives. Cooking was also sometimes used to facilitate bonding between families or friends.

Well my mam and dad always done cooking with me, like when I was younger. My mam and dad, my dad cooks all the time. Even down to where they showed me how to do rabbit stews. PARTICIPANT 10

I love baking my cakes… More so if I’ve got…if I’ve just got my girls in, weekend… Because it involves them, you see. PARTICIPANT 12

The reported cooking skills varied widely between participants, from no practical ability, to the capacity to prepare complex meals entirely from scratch. Participants who were interested in cooking often sought out opportunities to improve their skills, and were prepared to accept culinary failures along the way, whereas those with less engagement viewed their lack of ability as a significant barrier. Greater confidence with cooking was often associated with higher levels of skill. However, confidence was also influenced by the expectations associated with sharing meals, with variation in the perceived differing standards required for preparing food for oneself, partner or family, guests, and formal occasions.

When I’m cooking for other people I worry about it more. It was actually quite a stressful aspect in the last relationship I was in because I felt under pressure to produce a good meal pretty much every night for my girlfriend, as well as for myself. PARTICIPANT 4

For most participants, the process of cooking was strongly linked to their perception and experience of mealtimes. These included usual meal patterns and deviations; planning ahead for meals; and treats and rewards.

I have a cooked breakfast when I go away somewhere, just as a little treat… But I don’t have a cooked breakfast at home. Never, never. PARTICIPANT 5

Many people seemed to operate a mealtime ‘norm’, for example home cooked dishes shared with their family, which was modified according to competing demands, such as the time constraints imposed by others’ schedules.

*…and my partner also works shifts*… *He’s on early on a Thursday morning so I know if I’m getting in late on a Wednesday I know I need to have something made quickly*. *PARTICIPANT 2* (see photograph 6)

### Situational drivers

Participants frequently described how home food preparation behaviour was influenced by their mood and levels of energy and enthusiasm at a specific point in time. For example, preparing complex, time-consuming meals was generally more common at weekends than during the working week, since participants often felt pressured and tired after a day at work. Prompted by her own photograph, one participant described how:

*I make these [meals] up and put these in the freezer*, *in silver dishes*. *I take [them] out each day*. *PARTICIPANT 10* (see photograph 7)

Levels of motivation in specific meal situations could also vary greatly within the same participant in the short term from day-to-day.

So we all quite like eating and making food, but it’s usually just because when you get in you’re tired and you can’t really be bothered sometimes, but on weekends it’s different. PARTICIPANT 2

It’s just spur of the moment. If I’m in the mood for cooking then I’ll just do batches of cooking… If I’m not in the mood then I don’t do it. PARTICIPANT 11

Strategies used by participants to manage low enthusiasm for cooking involved short cuts to minimise time input and simplify food preparation, for example using pre-chopped vegetables.

*When I get home I’m tired so I don’t really want to cook for as long or prepare as long*, *so it’s usually quite fast dinners that I make*.. *PARTICIPANT 1* (see photograph 8)

Sharing meals and preparing them for others was a strong situational driver, with the levels of compromise reached varying between participants. With regards to scheduling, some participants prepared meals more quickly, or to fit in with others’ timetables, for example using pre-prepared ingredients rather than cooking from scratch; whereas others chose to eat separately. In terms of balancing food preferences, some participants perceived these as fixed parameters, preparing different dishes or meal variations according to the likes and dislikes of the household. Others viewed the situation flexibly, for example considering that children should be encouraged to diversify their tastes and eat the food served.

Yeah, so I usually eat it [dinner] with my boyfriend, but he… I am very fussy and he is very fussy, so we tend to have different foods. PARTICIPANT 1

I know with my friend whose a vegetarian, if she’s coming obviously I need to do vegetarian food… So to make it easier I will make something for all of us, rather than doing two separate meals. I just don’t tell them. PARTICIPANT 10

The sociability of preparing food for others provided an incentive to cook. Some participants described maintaining a supply of home cooked foods available in case guests should visit. Entertaining people for a meal also often influenced behaviour, both in terms of preparing more elaborate dishes, and eating in a more formal context. One participant, prompted by their photograph, noted:

*Oh*, *this is dinner at the table*, *which is Sunday*, *because we had someone around*, *and everything we served from dishes rather than serving straight onto the plate*, *which is what would normally happen*. *I would normally just serve onto the plate and then we would eat in the lounge*, *usually*, *on a lap tray or something like that*. *PARTICIPANT 2* (see photograph 9)

Participants living alone sometimes noted that preparing a meal for only themselves reduced their sense of engagement with cooking and seemed purposeless and time inefficient, which discouraged extensive food preparation.

I think it would be if I lived with someone, or in a family, or in a group of people, even a commune or something like that, where there was a focus on it [cooking] which I could join in with. That would encourage me to do a lot more, actually. PARTICIPANT 9

In contrast, some participants stated that living alone drove them to cook out of necessity.

*So when I got married my wife was a very good cook*, *and she did all the cooking*, *and it’s a bit sexist*, *really*, *I just let her do that*, *and she was happy to do it… She enjoyed cooking*. *And then when we separated I had to learn to cook*. *PARTICIPANT 5* (see photograph 10)

### Resources

Resource availability over the life course, in terms of time, money and facilities, was described by participants as an influence on their home food preparation behaviour.

#### Time

Some participants reported time as a limiting factor in their home food preparation. This was due to pressures both from themselves, such as their employment schedule, and other people, such as children’s extra-curricular activities. However, responses to this constraint varied widely. Some people avoided cooking by consuming ready meals, eating out and ordering take-aways; others greatly restricted their time allocation to cooking by using pre-prepared ingredients. Some participants maintained food preparation as a priority, for example cooking at weekends and freezing meals for later in the week; planning ahead extensively; and purchasing time-conserving cooking equipment.

Like on a Tuesday me and my partner both work late and the kids are at clubs so we all don’t get in until about seven o’clock, half past seven… So we would have a late tea then. Normally that’s something I would have in the slow cooker, or it would be one of the meals I’ve already had cooked so I can just make that. PARTICIPANT 10

Participants’ perceptions of time spent cooking also varied; some viewed cooking as another potentially stressful chore to be completed as quickly as possible, whereas others considered it an enjoyable use of time, for example marking the transition from work to home life, or demonstrating love and care in their role as provider and nurturer. Accordingly, participants who took pleasure in cooking were much less likely to perceive and cite time as a practical barrier to food preparation, and tended to spend longer cooking.

*Sundays*, *I always spend Sunday batch-cooking*… *Sunday afternoon*, *I quite enjoy it*. *PARTICIPANT 10* (see photograph 11)

#### Money

Most participants considered the cost of food in their decision making around cooking, though the context differed according to their financial situation. For example, some participants budgeted on food to ensure there was enough to feed them until the end of the week, whereas others deliberated whether the extra expense of premium products, such as organic goods, was justified.

*I work part-time*, *so my income’s not enormous*, *so I do think quite a lot about where I can get the cheapest food*. *PARTICIPANT 4* (see photograph 12)

Participants seemed divided on whether home cooking was more or less expensive than alternatives such as pre-prepared foods and take-aways, though were in general agreement that eating out was an expensive luxury.

Well, I did think that it is cheaper to get a takeaway instead of making a big massive thing of something, but I think well, if I do a big massive thing like you say, you could freeze it for next week, so that’s what I’ve started doing. PARTICIPANT 14

We don’t make a choice and say let’s go and eat out tonight, I don’t tend to do that, unless it’s a special occasion… I always think I can cook better value when I’m eating out… What you pay these days, actually, it’s ridiculous. PARTICIPANT 5

#### Facilities

For some participants, cooking facilities had a strong bearing on their approach to preparing food at home, with limited resources acting as a deterrent to cook.

But some days I just walk in [to the kitchen] and think ‘Agh’, and I’m like, ‘right pass the phone and we’ll order the Chinese’. But I think once it’s decorated I think I’ll be using it a lot more than what I am at the moment. PARTICIPANT 11

Yeah, that can make things really difficult when you don’t have the equipment and the kitchen that you need. PARTICIPANT 15

In contrast, participants also reported that cooking equipment could enable them to optimise their time and help fit cooking into a busy schedule.

My slow cooker, I couldn’t live without my slow cooker now because I just put it on. I chop all my veg on a night time. Put it in in the morning. I have everything ready, stock and everything ready, put it all in and I know when we come in at five, six o’clock it’s ready. PARTICIPANT 10

## Discussion

### Main findings

We conducted qualitative interviews with adults from varied socio-demographic backgrounds to provide insights into their practices, experiences and perceptions of home food preparation. Most people developed a personally satisfactory day-to-day coping approach, although preparing food was a compromise between diverse motivations and demands on resources. Driven largely by social desirability and a wish to identify themselves as a proficient cook, many participants aspired to increase their cooking from scratch, and to prepare healthier meals.

Our research highlighted home food preparation as a practical process and skill, with short-term situational drivers, and influenced by longer term facets of identity (see Fig 1). These three main themes were divided into two categories, namely personal motivation, for example enjoyment and engagement with cooking; and the influence of others, such as their food preferences. These factors interacted with each other, according to their salience and modifiability. For example, enjoyment of cooking helped participants to overcome potential barriers, such as family food preferences. Participants also noted the significance of resources for home food preparation, in terms of time, money, and facilities.

### Strengths and limitations

In contrast to previous research exploring home food preparation,[21, 36–38] we studied participants from wide-ranging socio-demographic backgrounds, rather than focussing on a particular subgroup. This highlighted the cross-cutting nature of key themes traversing the socio-demographic spectrum. All participants were recruited from the North East of England, hence their views may not be more widely generalisable. However, our findings reflect previous research emphasising the importance of factors such as time,[39] skills,[40] and shifts in behaviour at key transition points in life,[41] suggesting the main themes identified are likely to be transferable.

Our interview topic guide (S1 Appendix) was informed by a recent extensive systematic review of the determinants and outcomes of home cooking.[18] It is likely that this guide prompted consideration of relevant wide-ranging issues, and the use of open-ended questions ensured the generation of rich, detailed data. We conducted our interviews to reach thematic saturation, and there were no overall differences in the key themes identified from single interviews and those where other participants were also present.

We used photo-elicitation to successfully generate prompts to in-depth discussion.[42, 43] Visual methods,[44] particularly participant-generated photographs,[19, 45] help elicit detail from nuanced personal experiences. Participants maintained control over their research involvement, thereby avoiding bias against individuals with busy lifestyles, or limited cooking facilities, and promoting participant recruitment and retention. Multiple photographs provided data on a wider range of scenarios than a single observed cooking session, and may therefore more accurately reflect usual behaviour. The great majority of participants engaged effectively with photo-elicitation, and the variation in their submissions reflected different styles of telling their personal story of home food preparation. However, other methods such as go-along interviews[46] or ethnographic observation with think aloud interviews[47] might have offered further insights.

Both the professional and personal characteristics of an interviewer may impact on qualitative data collection, and its subsequent interpretation.[48] In order to reduce this likelihood, and the possibility that participants would provide socially desirable responses, we used a reflexive interviewing approach. This involved considering the interviewer’s perspective on interpretation of the findings; providing adequate time for participants to consider their responses; reminding them that honesty was more valuable than any perceived ‘right’ answer; and promoting full comprehension of all questions by rephrasing as necessary. In order to reduce potential bias in the analytical process, several different members of the research team conducted independent coding of transcripts and met regularly to cross-check the interpretation of key themes.

### Relationship to previous research

We identified the importance of considering multiple dimensions of home food preparation (see Fig 1), whereas previous qualitative studies have largely focussed on single aspects of cooking,[18] such as the influence of culture,[39] or impact of marriage and cohabitation.[41] Our results support findings from the United States[22] and island of Ireland[49] regarding the individuality, complexity and social importance of cooking. This study additionally highlighted changing patterns in food preparation behaviour according to varying demands and priorities over the lifecourse, and generated personalised insights into cooking attitudes and practices through the process of photo-elicitation.

Research into home food preparation has often concentrated on barriers, such as limited resources, and sought to explore constraints without explicitly considering that participants may be content with their current practices.[20, 40] In this study, individuals often stated that additional resources would be beneficial, however participants engaged in varying types and degrees of involvement in home cooking, throughout the spectrum of resource availability. This suggests that resources may have been used as a perceived socially acceptable response, whereas personal motivation and the influence of others generally determined the extent to which participants cooked. Hence interventions targeting resources alone may not result in comprehensive changes to home cooking behaviour. Furthermore, the impact of cooking interventions more broadly may be limited if people consider that their own cooking is acceptable, and that they would not benefit themselves from an intervention.

Much previous research has described the impact of busy lifestyles on time available for food preparation at home.[20, 50–52] We found no clear correlation between time availability and willingness or ability to cook, but rather the perception of time as a barrier to cooking was related to participants’ underlying opinion of themselves as a cook (identity), enjoyment and engagement with preparing food (process of cooking) and levels of energy and enthusiasm (situational drivers) (see Fig 1). Participants tended to learn to manage their cooking within the time available, and to devote more time if they experienced cooking as pleasurable and a priority, rather than a domestic chore.

We identified concurrent significance of both personal motivation and the influence of others in determining home food preparation practices. This extends previous research showing that older women,[53] older men,[54] and younger men[55] living alone all tended to experience challenges to preparing and eating wholesome meals. Similarly, our research found that preparing a one-person meal often provided little incentive to cook. However, in contrast, single people frequently noted the necessity to cook in order to fend for themselves.

### Implications

Our findings suggest that the most effective opportunities for intervention in home food preparation practices are likely to occur at transition points in life when incentives and circumstances for cooking change, such as leaving the parental home; commencing or ending cohabitation; adopting caring responsibilities; and retirement. Evidence from other domains, such as smoking cessation in pregnancy,[56] dietary changes following a cancer diagnosis,[57] and sustained weight loss after a personal crisis,[58] support the notion of ‘teachable moments’[59] or significant life stages for potential adoption of new health behaviours. Cooking interventions delivered at such transition points may therefore prove fruitful for changing food preparation habits and developing closer engagement with food and cooking.

Given that participants described making changes to their cooking behaviour, practices are generally modifiable, thereby presenting opportunities to create more conducive environments for preparing food at home. For example, policies could support initiatives for subsidising cooking equipment, or ensuring that adequate kitchen facilities form part of mandatory criteria for new properties and public or social housing.

However, our finding that many people establish home food preparation practices as a personally acceptable compromise between competing demands, indicates there may be a natural limit to the impact of cooking interventions. Approaches may therefore need to appeal to people’s reported aspirations to change. Tailored marketing could focus on adjusting social norms and personal priorities to promote a positive view of time spent in food preparation, in contrast to marketing campaigns against cooking.[60] This could include emphasising the accessibility of cooking, in contrast to complex, seemingly unachievable creations frequently portrayed in popular media. Strategies could also highlight the health significance of cooking for disease prevention and management, and the importance, as a responsible provider, of cooking for dependents.

### Future research

Our findings suggest that life transition points are important in determining home food preparation behaviour. Hence future research should involve longitudinal studies with duration encompassing key life changes, such as starting or ending cohabitation, taking on significant caring responsibilities, and retirement. Detailed questions on home food preparation could be incorporated into existing large-scale longitudinal surveys, which would enable exploration of key determinants and outcomes of home food preparation, and relationships with significant transition points in life. The successful use of photo-elicitation in our interviews to generate key insights indicates this is a promising strategy for use in future qualitative studies.

## Conclusions

In a study exploring home food preparation practices, experiences and perceptions, we identified the importance of both personal motivation and the influence of others. Key themes emerged regarding identity; the process of cooking; situational drivers; and resources. Home food preparation behaviour was often a balance between varied competing influences and demands in life. Overall, people were largely content with their cooking compromises; however many expressed an aspiration under ideal conditions to cook at home more often, using basic ingredients. Approaches to cooking varied greatly between individuals, and evolved in the short and longer term within the same individual, according to changing priorities and circumstances. These life transition points may prove effective junctures at which to offer support and interventions to encourage home food preparation. Interventions should be targeted at encouraging personal motivation and a shift in social norms, in order to prevent ambivalence regarding changes in behaviour. Longitudinal research studies to help establish causal relationships between the determinants and outcomes of home cooking over the lifecourse are also required.

## Supporting information

S1 Appendix Interview topic guide. Iteratively developed topic guide, with questions related to practices, experiences and perceptions of cooking.
(DOCX)

S2 Appendix Participant photographs. Photographs illustrating quotations provided in the main manuscript text.
(PDF)
