# Supplementary material for: Home food preparation practices, experiences and perceptions: A qualitative interview study with photo-elicitation
Source: PLoS One. 2017 Aug 30;12(8):e0182842. doi: 10.1371/journal.pone.0182842 (PMC5576640; doi:10.1371/journal.pone.0182842)
Supplement: S1 Appendix — Iteratively developed topic guide, with questions related to practices, experiences and perceptions of cooking. (DOCX) [file pone.0182842.s001.docx]

**Interview topic guide**

**Introduction**

Interviewer introduces themselves, and the aims of the study. Ground rules:

- Participant is free to state at any time if they feel uncomfortable with questions or want to stop the interview.
- The interview will be audio recorded and the interviewer will make brief notes. Both will be anonymised after the interview.
- There are no right or wrong answers and all responses are valid.

**Prompts**

- Tell me about the meals that you’ve had over the past week. Did you cook any meals? What did you prepare?
- Tell me about your cooking photos.

**General questions**

- Tell me about your eating habits and your usual home food preparation behaviour.
- What does home cooking mean to you?
- How do you feel about cooking (eg enjoyable, a nuisance)
- Are there particular aspects that you do or don’t like? Why?
- How would you describe yourself as a cook? (eg good, bad, OK, boring, safe, adventurous, nervous, healthy, unhealthy)
- Are you a confident cook? Why/why not?

**Household**

- Who prepares meals in your household?
- How often does the household have a cooked meal?
- What sort of meals do you have? (favourites/last night)
- During a typical week, how often do you cook a meal from scratch? (for example using raw chicken, spices and vegetables to make a chicken curry)
- During a typical week, how often do you cook a meal using pre-prepared ingredients? (for example using dried pasta and ready-made sauce)
- During a typical week, how often do you have ready meals and/or takeaways?
- During a typical week, how often do you skip meals or have snacks instead?
- What factors influence these choices?
- Which main dish do you consume most often? (to inform phase 3 analysis)
- Where do you eat your meals? Why?
- Do you eat with anyone else? Who and why?

**Determinants**

- What influences your eating habits?
- What factors make it easier for you to cook? (eg lots of time, good kitchen facilities, enjoyment)
- What factors make it difficult for you to cook? (eg too busy, ingredients are expensive, don’t have necessary skills)
- What factors would you like to change?
- Who has an impact on what and how you cook?
- Has your home food preparation behaviour changed over time? Why/why not? Would you like to change in the future?

**Health**

- What does healthy cooking and eating mean to you?
- Does the healthiness of foods influence your cooking and eating patterns?
- What comes before health in your priorities and why?

**Skills, facilities and shopping**

- What cooking and storage facilities do you have? (eg hob, fridge, cupboards)
- What utensils and equipment do you have? (eg pans, knives, chopping board)
- Do you have any cooking skills? If so, which? (show card of different skills eg frying, grilling, roasting)
- Where did you learn? (eg mother, school, picked it up as you went along)
- Do you use recipes? Where from and why? Do you find them easy to follow?
- Do you plan your meals and/or food shopping in advance?
- Who does the food shopping and why? How often do you go shopping? What kind of shops/online? How do you decide what to buy?

**Wrap up**

- Is there anything else you’d like to mention that we haven’t covered?
- Close; thank the participant; and provide debriefing sheet and ‘thank you’ voucher.
