# Supplementary material for: Home food preparation practices, experiences and perceptions: A qualitative interview study with photo-elicitation
Source: PLoS One. 2017 Aug 30;12(8):e0182842. doi: 10.1371/journal.pone.0182842 (PMC5576640; doi:10.1371/journal.pone.0182842)

S2 Appendix Participant photographs

Photographs illustrating quotations provided in the main manuscript text.

1. Participant 9 typically cooked the same meal every night, comprising of filled pasta, a ready-made sauce, and boiled fresh vegetables, with slight variations. He described enjoying food, but not being interested enough in cooking to dedicate much of his time to preparing meals.

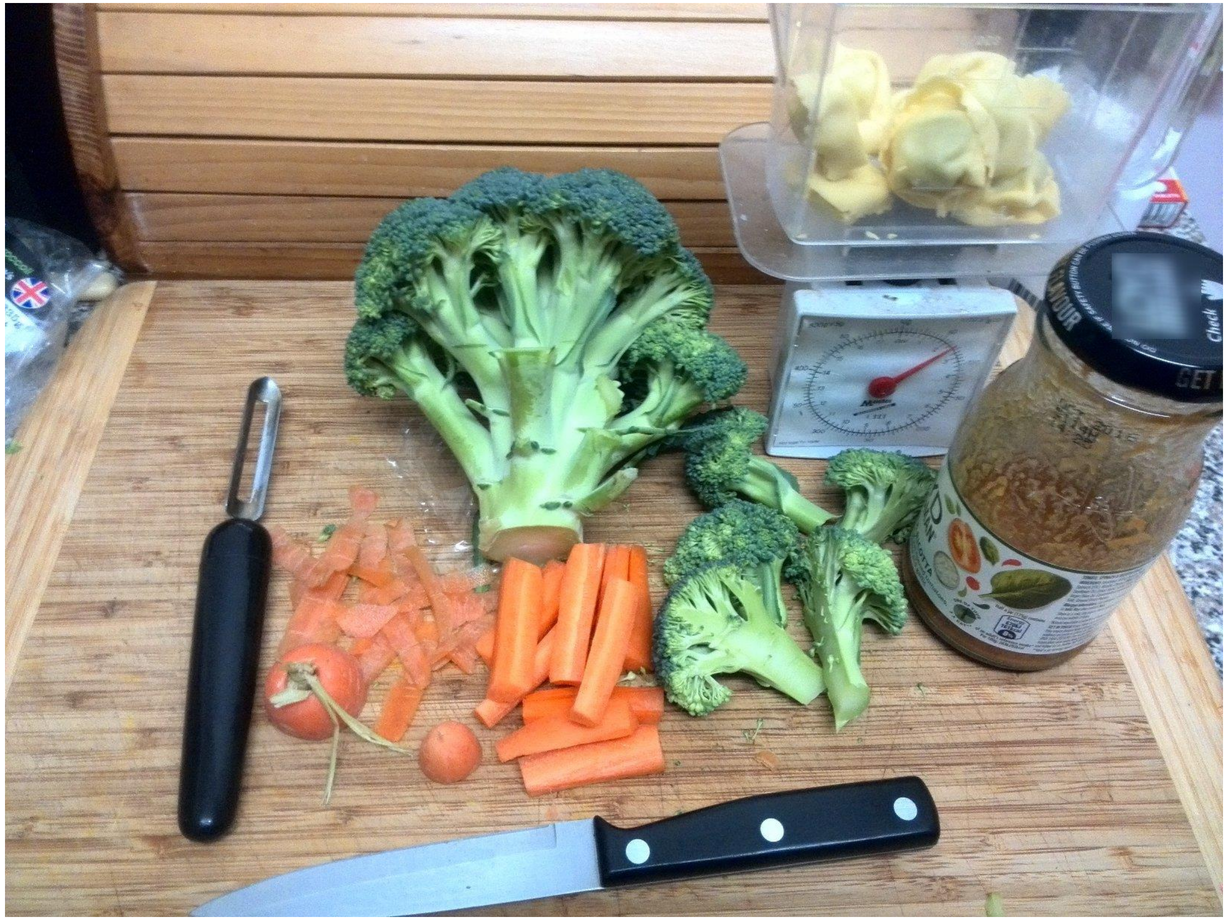

2. Participant 7 was a full-time housewife and prepared traditional Indian meals from basic ingredients several days of the week. She described her role in life as providing and caring for her family.

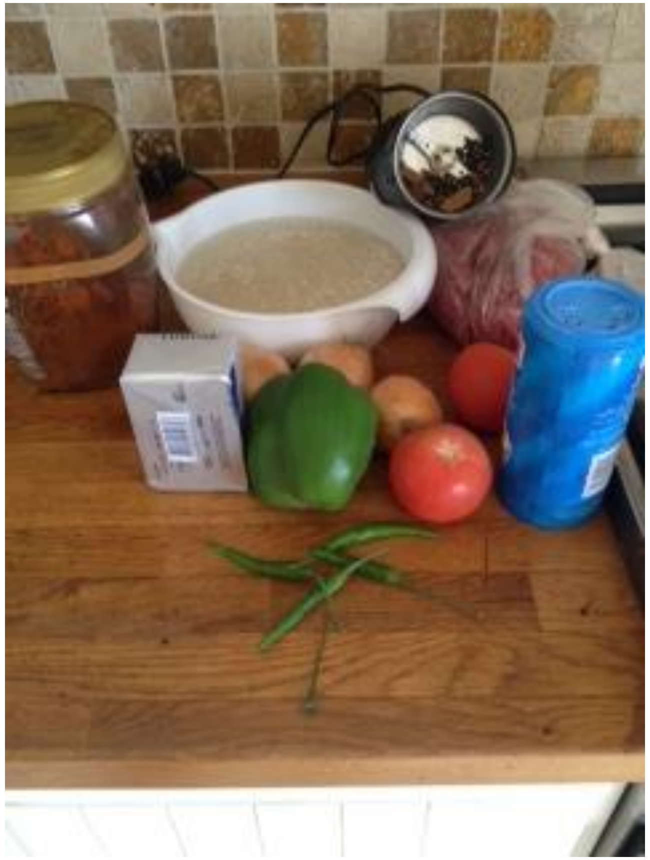

3. Participant 2 often purchased meal ingredients which were ready to assemble with minimal personal involvement. She described this as preferable to buying a composite ready-meal.

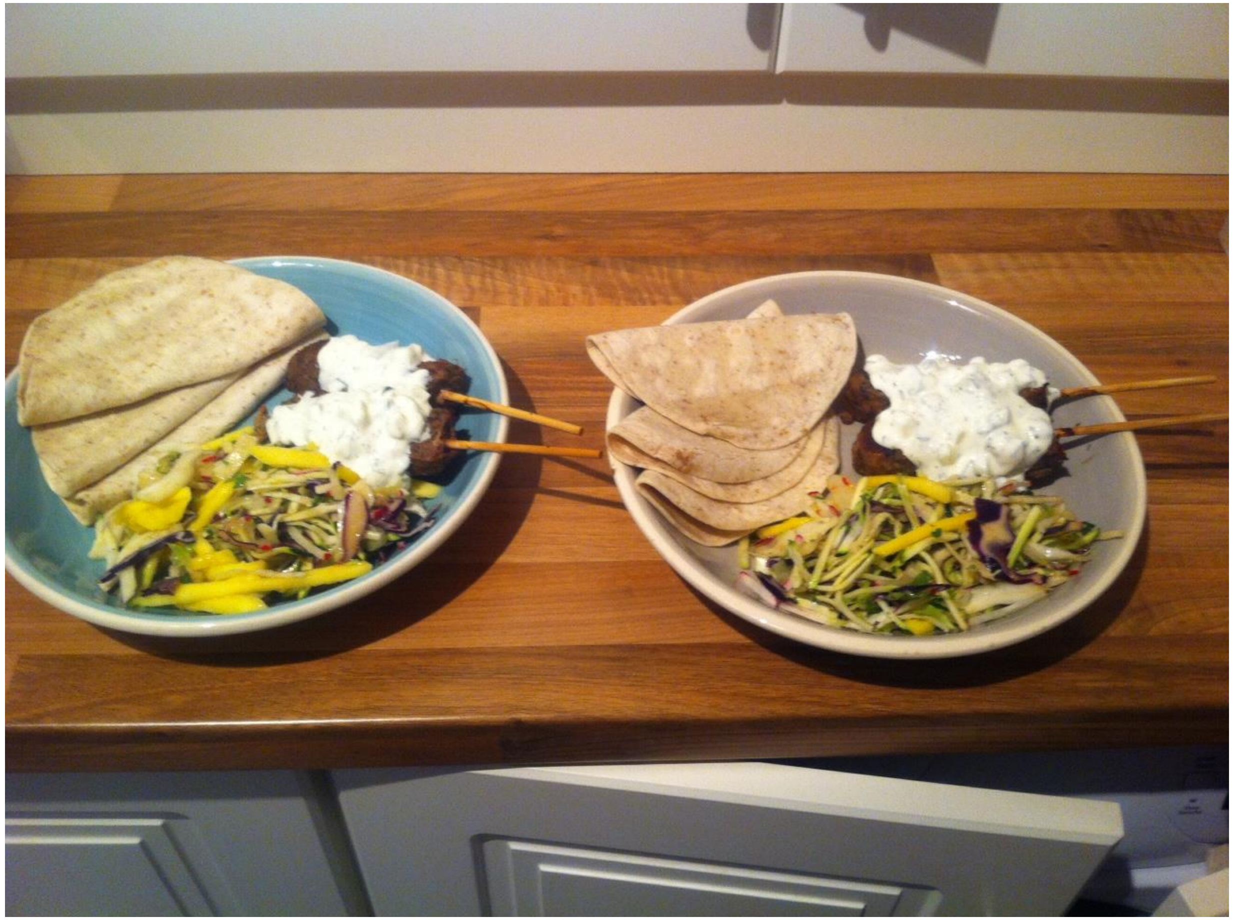

4. Participant 4 described taking an organised approach to meal planning, food shopping and cooking, which had evolved over time as he became more independent and self-sufficient.

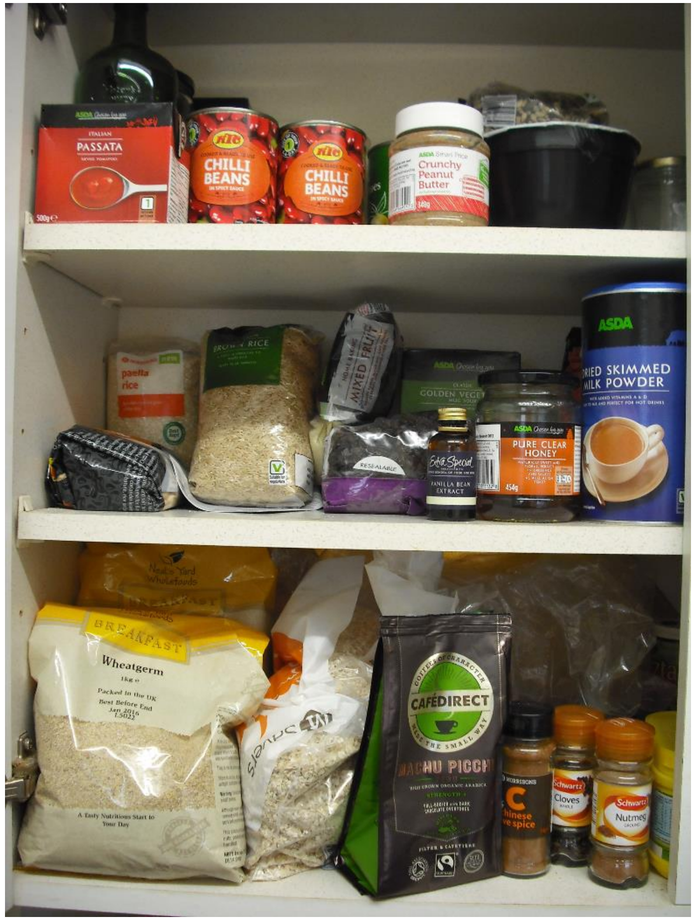

5. Participant 8 stated that he had no interest in cooking, and when eating at home consumed ready-meals, pre-prepared foods such as tinned soup, and sandwiches.

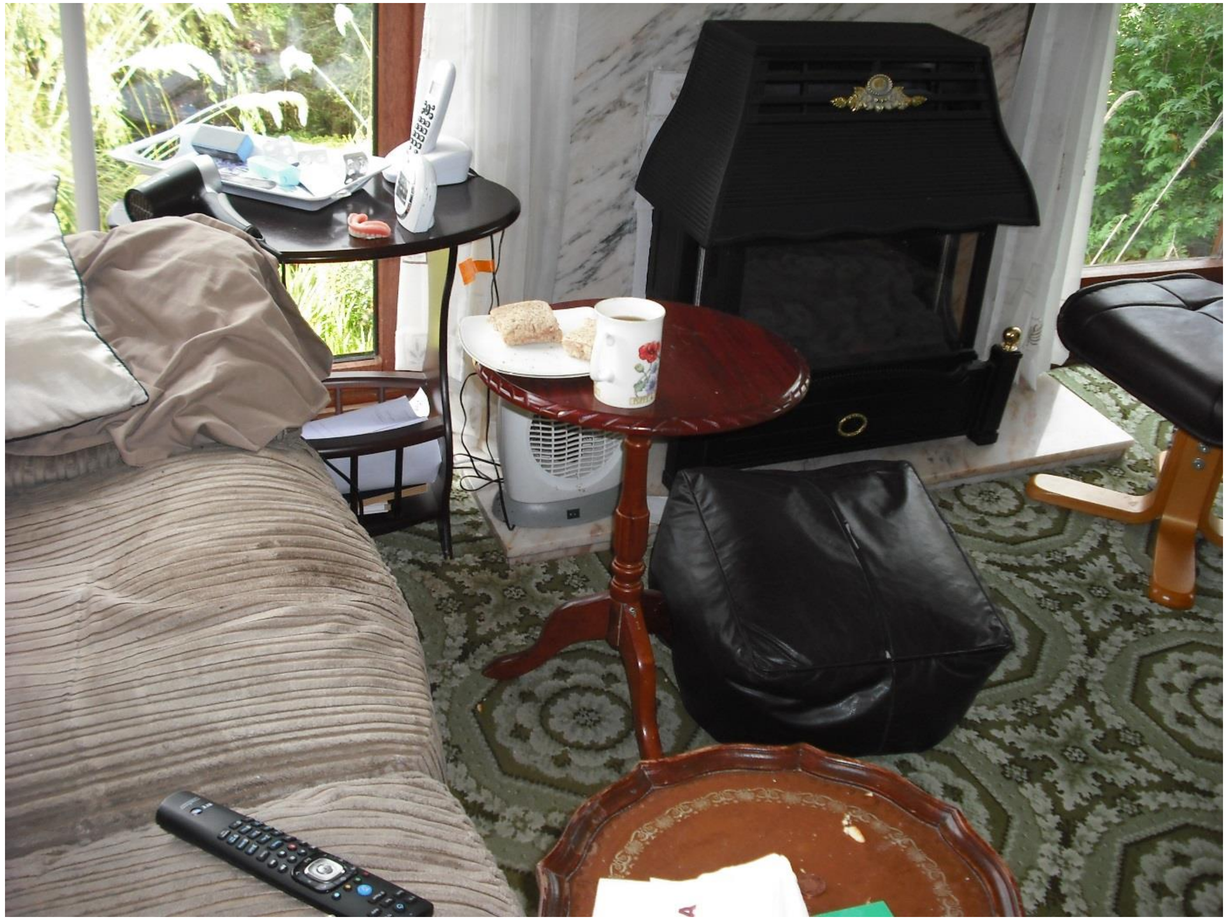

6. Participant 2 described preparing quick meals during the working week, such as omelettes, particularly when time was tight to fit in with her partner’s schedule.

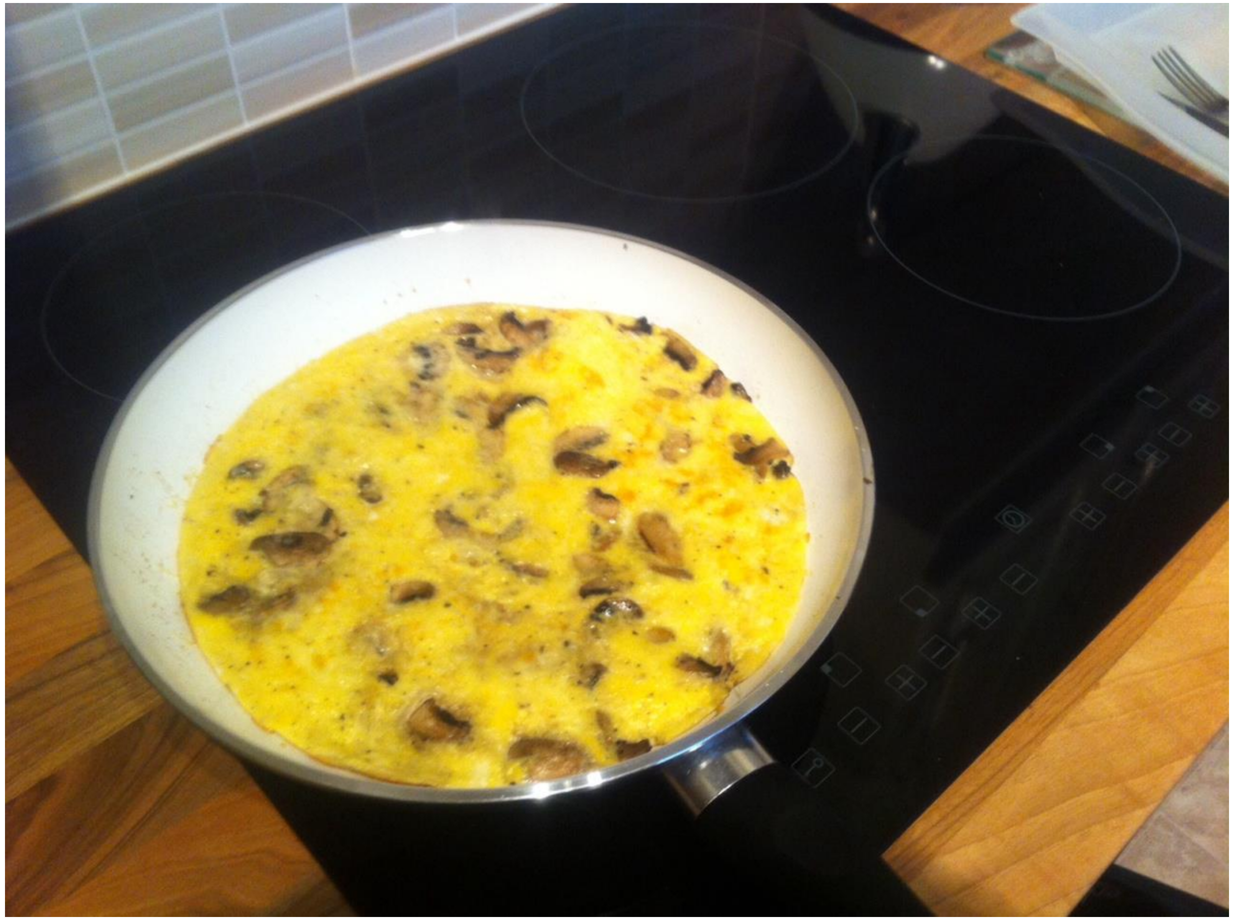

7. Participant 10 reported batch cooking at the weekends and then portioning up meals, ready for the busy week ahead.

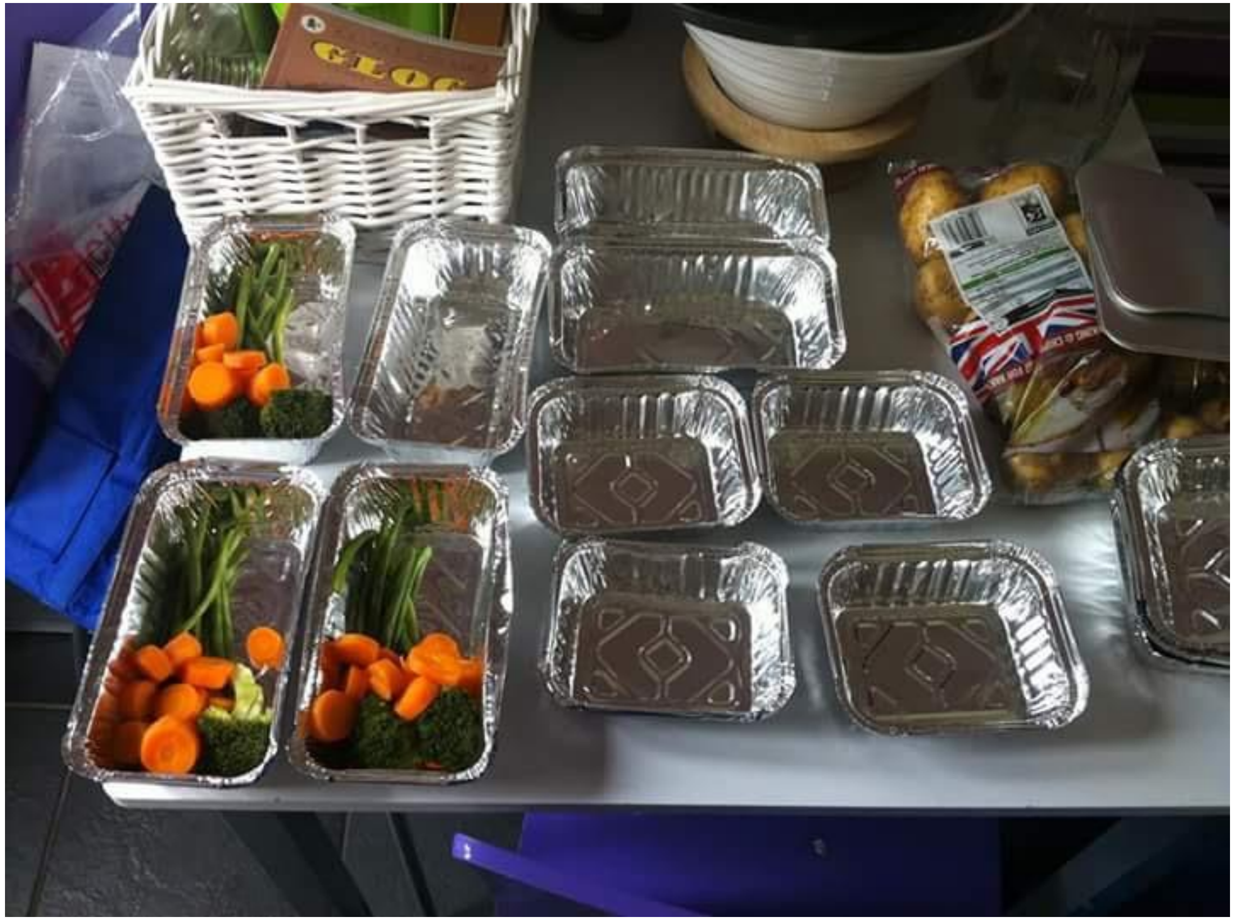

8. Participant 1 described how her enthusiasm and energy for cooking during the working week was often low, so she tended to prepare simple dishes, such as pasta.

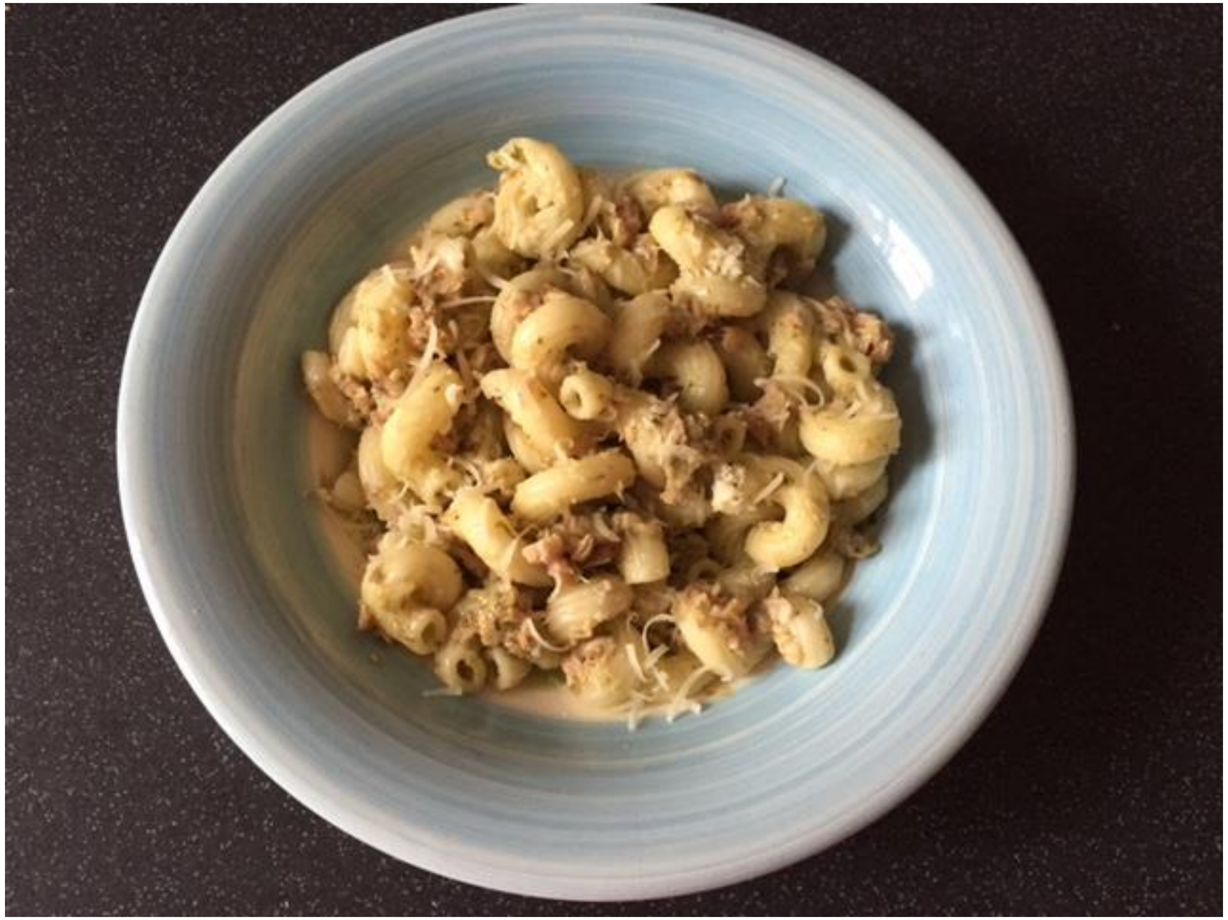

9. Participant 2 explained that the meal photographed was unusual in that it was consumed at a table, using serving dishes, whereas usually the meal would be served directly onto plates and eaten off a lap tray. The difference was due to a guest visiting to share the meal.

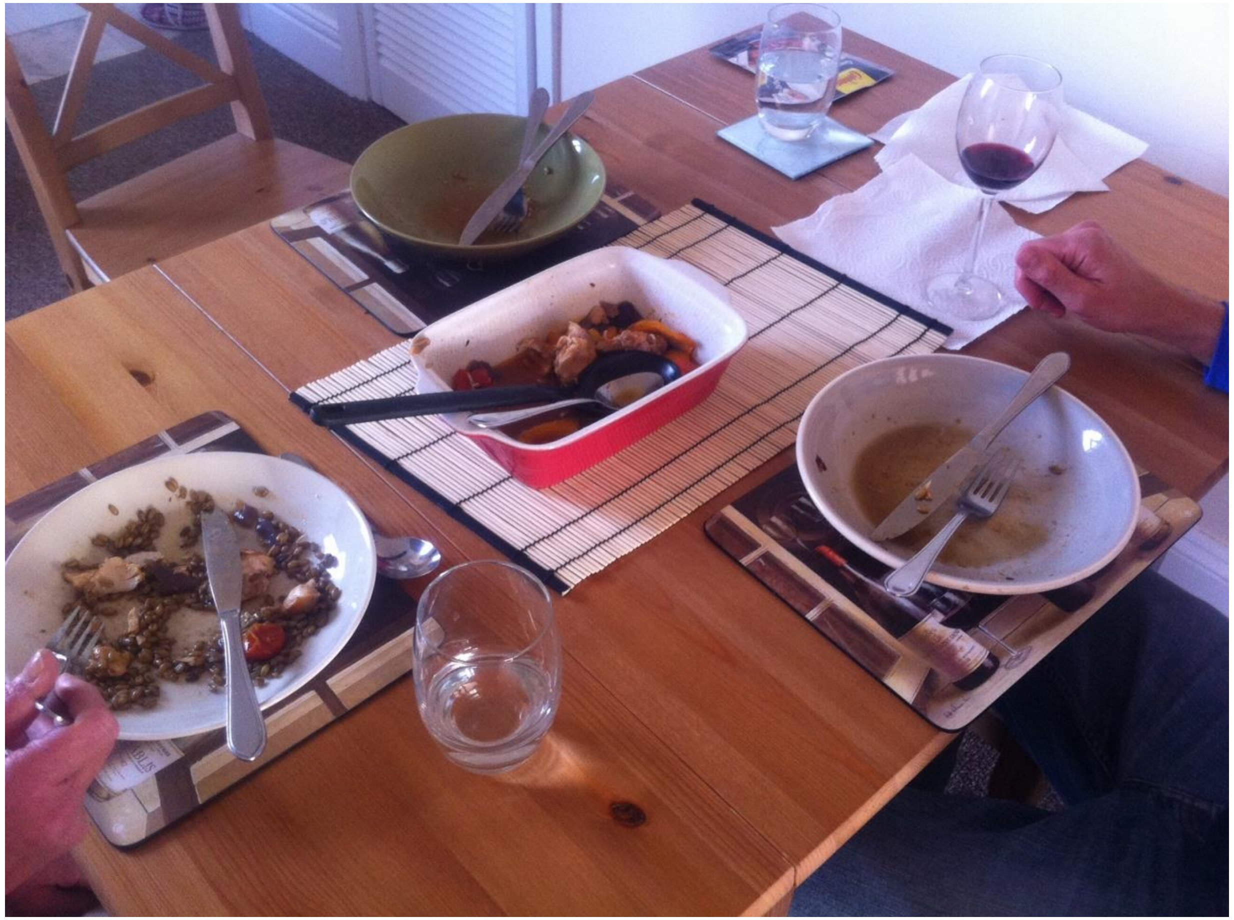

10. Participant 5 reported learning to cook out of necessity when he separated from his wife, initially using cook books.

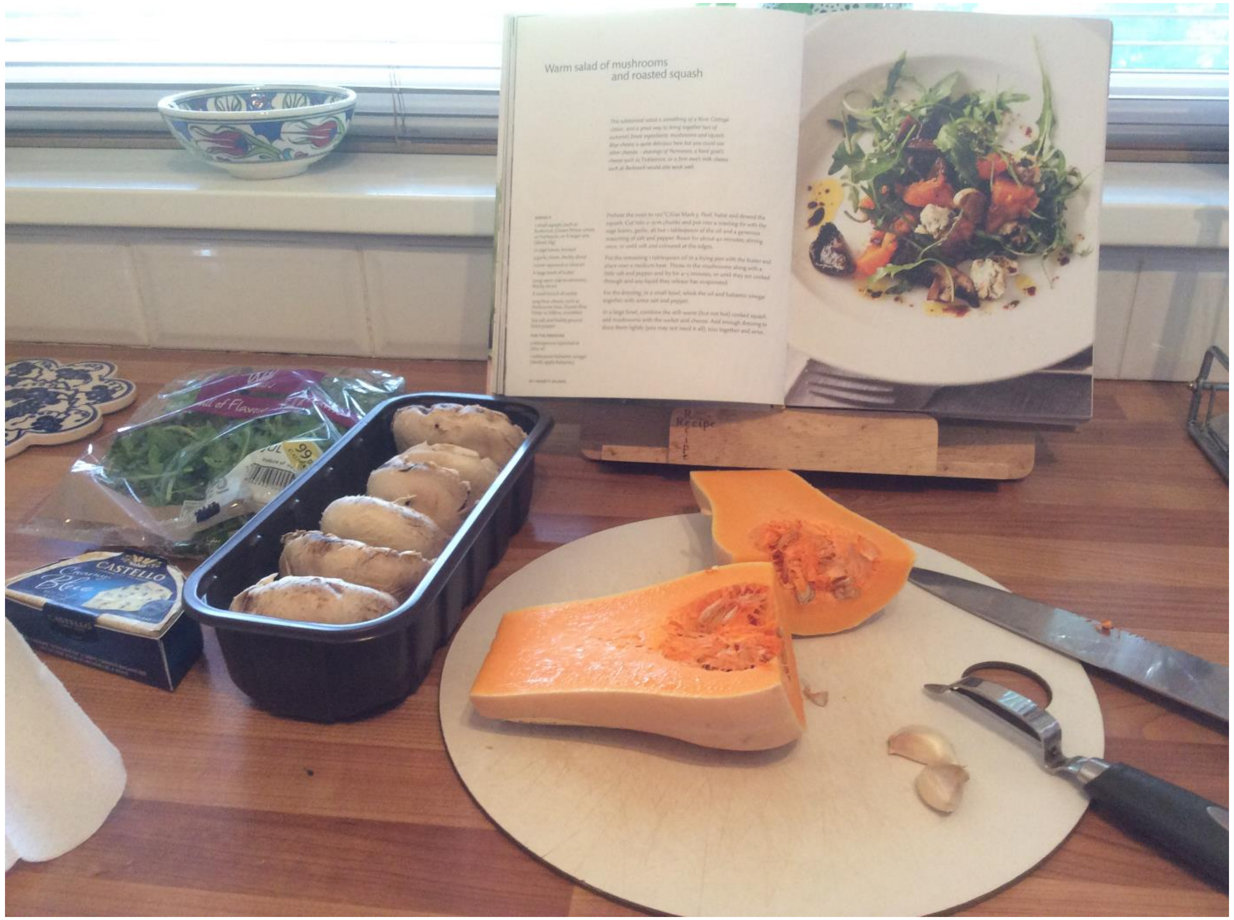

11. Participant 10 described how she tackled a shortage of time for cooking during the working week by preparing large quantities of food at the weekend, and then freezing it in batches. She found cooking enjoyable, and by taking this approach, lack of time ceased to be a barrier to eating home cooked meals daily.

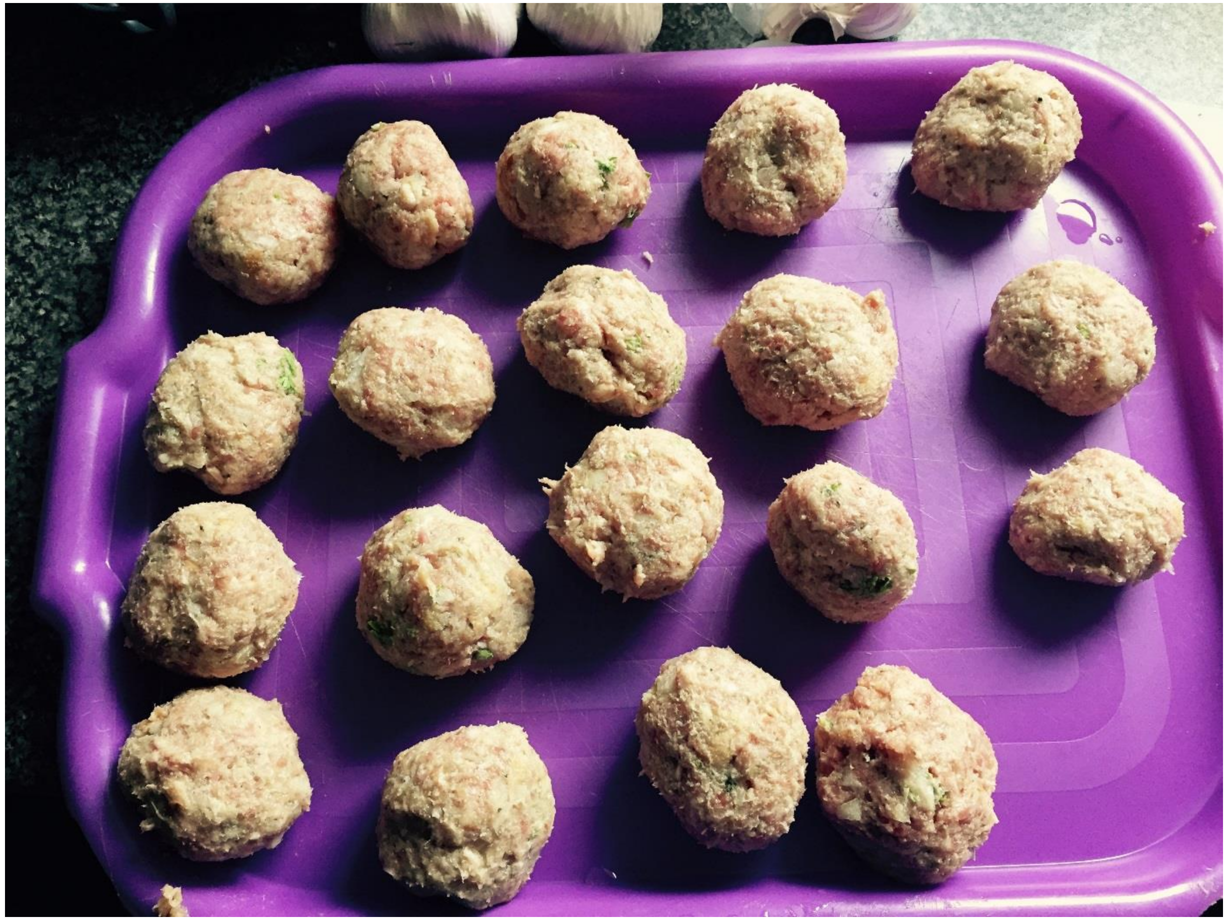

12. Participant 4 explained how he regularly visited a range of supermarkets to search for cut-price items, in order to reduce his food shopping bill.

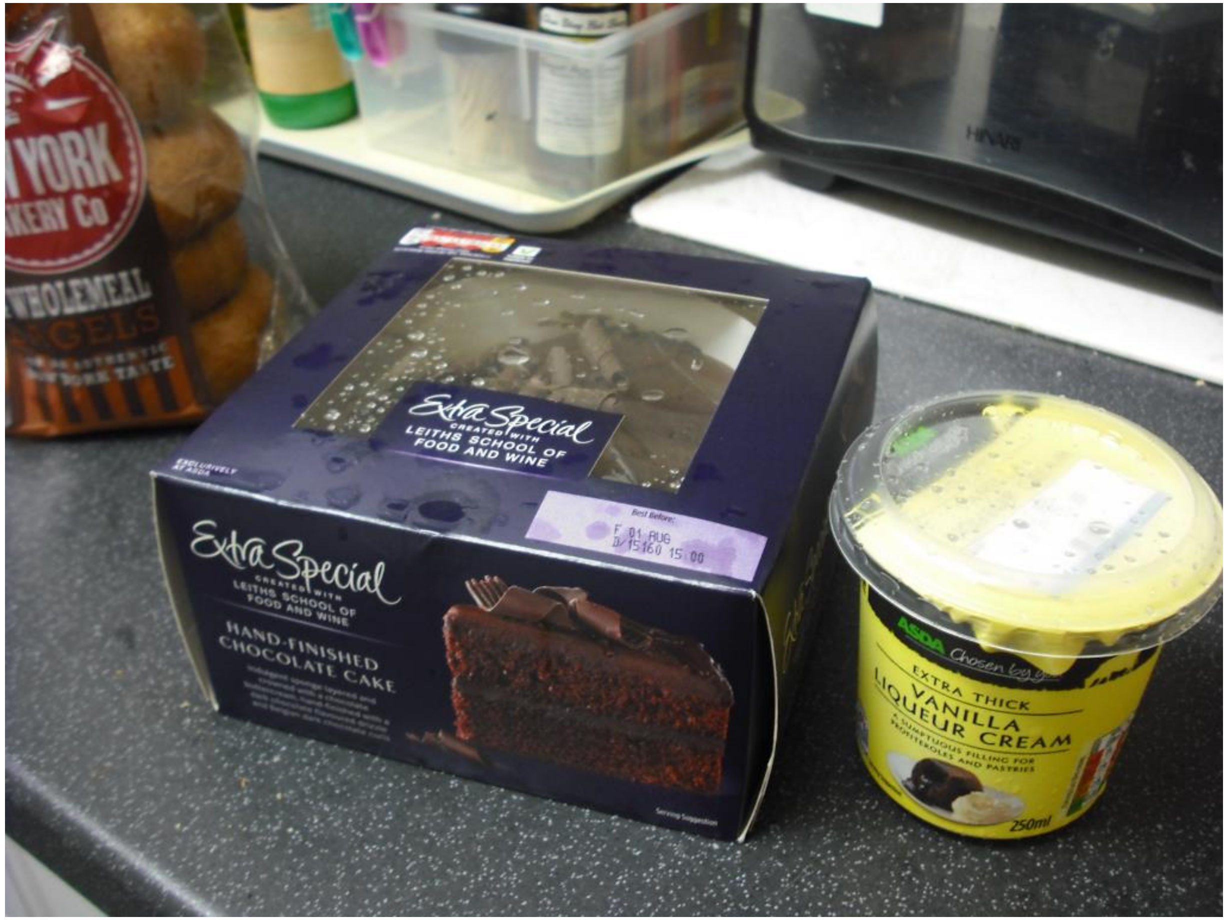

Supplement: S2 Appendix — Photographs illustrating quotations provided in the main manuscript text. (PDF) [file pone.0182842.s002.pdf]
